# The emerging role of anti-thymic stromal lymphopoietin monoclonal antibody (Tezepelumab) in comorbid and non-comorbid CRSwNP patients: a scoping review

**DOI:** 10.1016/j.bjorl.2026.101768

**Published:** 2026-01-17

**Authors:** Antonio Moffa, Eugenio de Corso, Domiziana Nardelli, Antonella Loperfido, Jacopo Galli, Peter Baptista, Manuele Casale

**Affiliations:** a Università Campus Bio-Medico di Roma, School of Medicine, Fondazione Policlinico Università Campus Bio-Medico, Integrated Therapies in Otolaryngology, Rome, Italy; b A. Gemelli Hospital Foundation IRCCS, Unit of Otorhinolaryngology-Head and Neck Surgery, Rome, Italy; c Università Campus Bio-Medico di Roma, School of Medicine, Rome, Italy; d San Camillo Forlanini Hospital, Otolaryngology Unit, Rome, Italy; e Catholic University of The Sacred Heart, Department of Head, Neck and Sensory Organs, Rome, Italy; f Al Zahra Private Hospital Dubai, ENT Department, Dubai, United Arab Emirates

**Keywords:** Tezepelumab, CRSwNP, Thymic stromal lymphopoietin, Biologics, Type 2 inflammation

## Abstract

**Objective:**

This systematic review aims to evaluate the effectiveness of Tezepelumab, a monoclonal antibody targeting the thymic stromal lymphopoietin, in patients affected by chronic rhinosinusitis with nasal polyposis, both with and without comorbid asthma.

**Methods:**

A systematic search of the literature was conducted across PubMed, SCOPUS, and Google Scholar databases until April 2025. Studies were selected based on predefined inclusion criteria, focusing on adult patients with clinically diagnosed chronic rhinosinusitis with nasal polyps who received Tezepelumab treatment for at least four weeks and reported validated clinical outcomes. Studies involving patients receiving prior standard treatment and comorbid asthma were included. Outcome measures included sinonasal symptom scores, imaging scores, pulmonary function tests, quality of life assessments, systemic corticosteroid use, and the need for surgical intervention. Study selection, data extraction, and quality assessment were conducted independently by two reviewers.

**Result:**

Out of 221 screened records, three randomized controlled trials involving a total of 691 patients met the eligibility criteria. Tezepelumab treatment resulted in significant improvements in nasal symptom scores, including reductions in nasal congestion and loss of smell, as well as improved quality of life and sleep-related symptoms. Objective assessments showed a reduction in polyp size and radiological scores, with fewer patients requiring systemic corticosteroids or surgical intervention compared to placebo. Improvements were also observed in lung function and disease control among patients with comorbid asthma. The effect size exceeded minimal clinically important difference thresholds across key outcome measures.

**Conclusion:**

Tezepelumab demonstrated consistent and clinically meaningful benefits in the treatment of chronic rhinosinusitis with nasal polyps, including symptom relief, reduced polyp burden, and decreased need for corticosteroids or surgery. Its upstream mechanism offers potential advantages over existing biologic therapies. Further long-term and real-world studies are needed to confirm its role in clinical practice and define ideal patient selection strategies.

## Highlights

• Tezepelumab improves CRSwNP symptoms and polyp burden.
• Tezepelumab reduces nasal congestion and loss of smell in CRSwNP.
• Tezepelumab is effective in patients with CRSwNP and comorbid asthma.
• Tezepelumab lowers systemic corticosteroid and surgical needs.
• Tezepelumab targets upstream inflammation, including T2 and non-T2 types.

## Introduction

Chronic Rhinosinusitis (CRS) is a multifactorial inflammatory chronic condition with two clinical phenotypes: CRS without Nasal Polyps (CRSsNP) and with Nasal Polyps (CRSwNP). While this phenotypic distinction remains formally useful, it is now recognized as an oversimplification. The complexity of CRS is better captured by its underlying endotypic drivers, which can cross canonic phenotypic boundaries and are more predictive of response to targeted therapies. CRS (with or without NP) has three endotypes: T1 is characterized by the type 1 cytokines interferon gamma and transforming growth factor beta; T2 by the type 2 inflammatory proteins and cytokines immunoglobulin E, Interleukin (IL)-4, IL-5, and IL-13; and T3 by the cytokine IL-17A produced by T-helper 17 cells.^1^ In T2 CRSwNP, eosinophils make up the predominant inflammatory cells in the mucous lining of the sinuses. However, type 2 innate lymphoid cells, B-cells, macrophages, dendritic cells, mast cells, and basophils are also found.^2^ Patients with the T2 endotype may also have increased fractional exhaled nitric oxide levels and either normal or elevated serum total immunoglobulin E.^3^ CRSwNP is characterized by the development of benign, oedematous nasal polyps originating from the ethmoid sinuses and extending into the nasal cavities.^4^

The prevalence and molecular profiles of CRSwNP display a considerable geographic variation. For instance, the prevalence of CRSwNP is approximately 1.1% in China, which is markedly lower than rates typically reported in European and North American populations, where it represents 20% of cases.^5^^,^^6^ Moreover, the distribution of inflammatory endotypes also differs greatly: although in the Western world about 80% of uncontrolled CRSwNP are characterized by a type 2 signature, this proportion ranges between 20% and 60% in China, Korea or Thailand, respectively.^7^ However, some patients with uncontrolled CRSwNP exhibit non-eosinophilic inflammation. Currently, monoclonal antibodies such as dupilumab, omalizumab, and mepolizumab provide new treatment methods for type 2 inflammatory CRSwNP. Biologics targeting type 2 immune effectors such as Interleukin (IL)-4, IL-5, IL-13, and free immunoglobulin E (IgE) have provided novel therapeutic options for patients with severe and uncontrolled CRSwNP.^8^ Thymic Stromal Lymphopoietin (TSLP) belongs to the Interleukin (IL)-2 family and participates in maintaining the stability of the mucosal immune system. TSLP is generally produced by keratinocytes located in the skin, epithelial cells in the intestine, upper airway, and lungs, Dendritic Cells (DCs), mast cells, and monocytes.^9^ The released TSLP stimulates the activation of immature DCs and presents allergens, thereby activating type 2 inflammation. A previous study demonstrated that the upregulated expression of TLSP in nasal polyp tissues and the expression levels of TSLP mRNA were positively correlated with the level of tissue eosinophils and the expression level of type 2 cytokines in CRSwNP patients.^1^

Additionally, bone marrow DCs activated by TSLP can promote the activity of non-eosinophilic inflammation. Elevated levels of TSLP appear to be associated with asthma and atopic dermatitis, which are typically T2 inflammation-mediated conditions. It is believed that the chronic overexpression of TSLP may result in an increased sensitivity to allergens.^10^ Moreover, TSLP could also be related to COPD pathogenesis.^3^ Initially, specific T2 cytokines such as IL-4, IL-5, and IL-13 have been studied to develop drugs able to inhibit or regulate their biological activity^15^; then, given the consolidated role of alarmins in epithelial-derived inflammation and the search for a possible alternative to the above-mentioned cytokines target, research was on drugs against alarmins, particularly TSLP.^3^ The first marketed drug against alarmins is Tezepelumab, an IgG2λ monoclonal antibody developed with the goal of modulating type 2, and to some extent, non-type 2, inflammation.^11^ This biological treatment can inhibit the action of TSLP by binding to it and preventing, in turn, its binding to its heterodimeric receptor TSLPR.^12^ Tezepelumab has recently been approved for the treatment of patients with severe asthma and is being studied for patients with other diseases such as CRSwNP and COPD.^13^^,^^14^ Trials on the efficacy of Tezepelumab not only demonstrate the expected efficacy in T2 patients but also, interestingly, a partial effect in patients whose inflammation is not definable as T2, which has been previously devoid of available biological treatments.^15^ The objective of this review is to summarize the anti-inflammatory effects and efficacy of tezepelumab for the treatment of patients with CRSwNP, including those with and without comorbid asthma.

## Methods

### General study design

The study was designed following the recommendations of the Centre for Review and Dissemination’s Guidance for Undertaking Review in Health Care and is reported in adherence to the Preferred Reporting Items for Systematic Review and Meta-Analyses (PRISMA) statement.^16^

### Data source and study searching

An electronic search was performed on the PubMed/MEDLINE, Google Scholar, and SCOPUS databases. An example of a search strategy is the one used for PubMed/MEDLINE: “Tezepelumab” and “Chronic Rhinosinusitis”; “Tezepelumab” and “CRSwNPs and “asthma”; “Tezepelumab” and “asthma”; “Tezepelumab” and “SNOT-22 score”; “Tezepelumab” and “SNOT-22”; “Tezepelumab” and “ACQ-6 score”; “Tezepelumab” and “AQLQ (S) +12 score”; “Tezepelumab” and “ASD score”; “Tezepelumab” and “Lund-Mackay score”; the searches were adjusted to fit the specific requirements for each database, with a cross-reference search to minimize the risk of missing relevant data. The last search was performed in April 2025.

### Inclusion/Exclusion criteria

We included the studies with the following characteristics: patients (P), adult patients affected by chronic rhinosinusitis with nasal polyps (CRSwNPs), defined by a Nasal Polyp Score (NPS) ≥5 (minimum score ≥2 for each nostril) and/or a Sinonasal Outcome Tests-22 (SNOT-22) ≥30, ongoing for at least 12-months and with comorbid asthma, whom have previously received stable standard care treatment for at least 30-days and have had documented treatment with systemic glucocorticoids in the previous 12-months and/or had been receiving medium- or high-dose inhaled glucocorticoids (daily dose of at least 500 μg fluticasone propionate or equivalent) for at least 12-months and at least one additional controller medication, with or without oral glucocorticoids, for at least 3-months and/or have had previous nasal-polyp surgery of any type; Intervention (I), at least 4-weeks of biological therapy administration (Tezepelumab 210 mg, one subcutaneous injection every 4-weeks) indicated specifically for CRSwNP treatment; Comparison (C), pretreatment and post-treatment; Outcome (O), Sinonasal Outcome Test 22 (SNOT-22), Lund-Mackay score, Forced Expiratory Volume in 1 second (FEV1), Asthma Control Questionnaire-6 (ACQ-6), Asthma Quality of Life Questionnaire (standardized) for patients 12-years and older (AQLQ[S] +12), Asthma Symptom Diary (ASD); and study design. The exclusion criteria were: 1) Studies not written in English; 2) Case reports, reviews, conference abstracts, and letters; 3) Studies with unclear and/or incomplete data; 4) Pediatric studies; and 5) Studies published before 2009 to discuss the solution of the last 15-years.

### Data extraction and data analysis

The articles were first screened based on their titles and abstracts. Next, the full-text versions of each publication were evaluated, and those deemed irrelevant to the review’s topic were excluded. Data extraction from the included studies was conducted systematically using a structured form. A qualitative synthesis was performed on the selected studies to analyze tezepelumab’s effects on sleep quality. We conducted a formal quality assessment of the included studies using the GRADE (Grading of Recommendations, Assessment, Development and Evaluation) tool. The overall quality of evidence was assessed as moderate. The main factors that influenced this assessment include:

• Inconsistency: Moderate heterogeneity in results across studies was observed, likely due to differences in study populations and measurement methods.
• Imprecision: Some studies had relatively small sample sizes, resulting in wide confidence intervals.
• Indirectness: Most studies assessed sleep quality as a secondary rather than primary outcome.
• Publication bias: Formal assessment of publication bias was not possible due to the limited number of studies.

### Statistical analysis and summary of findings

Due to the heterogeneous reporting styles and insufficient data in the included studies, conducting a statistical analysis or providing a quantitative summary of the findings was not feasible. Therefore, the effects on individual outcomes and the overall quality assessments were described narratively. The authors of the included studies were not contacted for additional information.

### Clarifying clinical relevance

To better contextualize the clinical relevance of the observed improvements in sleep quality, it is important to consider the Minimal Clinically Important Differences (MCID) for the measures used:

• SNOT-22 (sleep domain): The MCID for the total SNOT-22 score is well established at 8.9-points.
• FEV1: A change of 0.1 L from baseline in pre-bronchodilator FEV1 is generally considered clinically significant.
• AQLQ[S] +12: A change of 0.5 points in the AQLQ[S] +12 score (0–7 scale) is considered clinically significant.
• ACQ-6: A change of 0.5 points in the ACQ-6 score (0–6 scale) is considered clinically significant.
• ASD: A change of 0.5 points in the ASD score (0–4 scale) is considered clinically significant.

In the studies examined, treatment with Tezepelumab generally produced improvements that exceeded these MCID thresholds, suggesting that the observed benefits are not only statistically significant but also clinically relevant for patients.

## Results

**Fig. 1:**
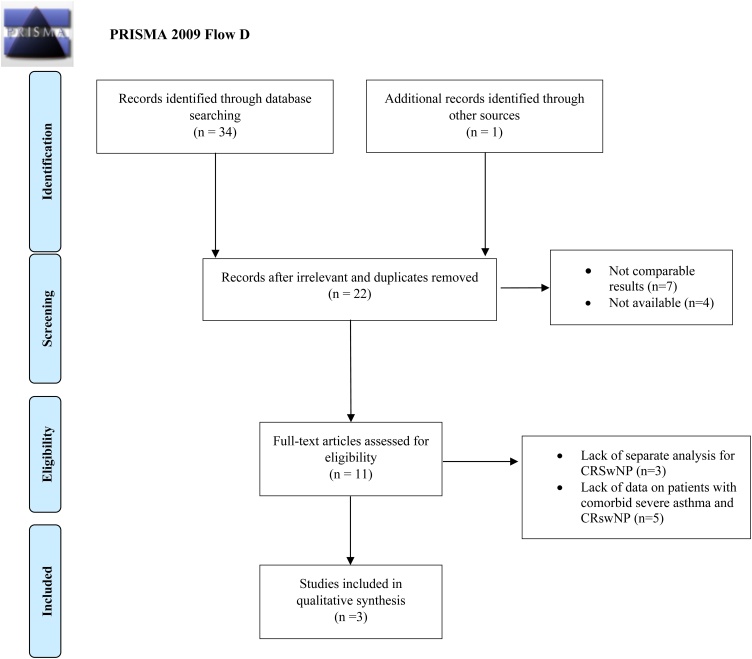
PRISMA flow diagram.

Although our initial search identified several studies investigating the effects of Tezepelumab in patients with comorbid severe asthma and CRSwNP, only 3 studies met our rigorous inclusion criteria. Studies were excluded primarily due to: 1) Absence of specific data on patients with both severe asthma and CRSwNP; 2) Lack of separate analysis for CRSwNP patients within mixed study populations. This stringent selection process, while limiting the number of included studies, ensured that the analysed data were specifically relevant to our research question. The selection process is illustrated in Fig. 1 (PRISMA flow diagram). The included studies involved 691 patients, with a mean age of 49.5-years. The characteristics of these studies are detailed in Table 1, with further descriptions available in Table 2.

**Table 1:** The characteristics of the studies included.

Table 1A Basic study characteristics					
Author (year)	Country	Journal	Study Design	Number of Patients	Follow-up
Jacobs et al., 2025	USA	Advances in Therapy	Phase 3, double-blind, placebo-controlled RCT	1059 (No history of CRSwNP: 894; History of CRSwNP: 165)	Week 28 and week 52
Lipworth et al., 2025	Canada, China, Denmark, Germany, Hungary, Japan, Poland, Spain, UK, USA	The New England Journal of Medicine	Phase 3, multicenter, parallel-group, double-blind, RCT	408	Week 52
Laidlaw et al., 2023	USA	Journal of Asthma and Allergy	Phase 3, double-blind, placebo-controlled RCT	1059 (No history of CRSwNP: 941; History of CRSwNP: 118)	Week 52

Table 1B Demographics and biomarkers						
Author (year)	Mean Age (years) (mean ± SD)	Sex (M/F)	Blood Eosinophils	Serum Total IgE	FeNO level (median)	Number of exacerbations in last 12 months
Jacobs et al., 2025	No history of CRSwNP: 48.9 ± 16.6; History of CRSwNP: 52.3 ± 12.3	No history of CRSwNP: 313/581; History of CRSwNP: 74/91	Median No history of CRSwNP: 240; History of CRSwNP: 425	Median No history of CRSwNP: 199.4; History of CRSwNP: 166.1	No history of CRSwNP: 29.0; History of CRSwNP: 45.0	No history of CRSwNP: 38.4% >2; History of CRSwNP: 53.4% >2
Lipworth et al., 2025	49.7 ± 13.6	266/142	Mean: 358 ± 238	Mean: 176.2 ± 284.7		
Laidlaw et al., 2023	No history of CRSwNP: 49.3 ± 16.4; History of CRSwNP: 50.8 ± 12.9	No history of CRSwNP: 334/607; History of CRSwNP: 53/65	Median No history of CRSwNP: 240; History of CRSwNP: 425	Median No history of CRSwNP: 199.4; History of CRSwNP: 166.1	No history of CRSwNP: 29.0; History of CRSwNP: 45.0	No history of CRSwNP: 38.4% >2; History of CRSwNP: 53.4% >2

**Table 2:** Summary results.

Table 2A SNOT-22 results						
Author (year)	SNOT-22 Total score (mean ± SD)	Nasal domain (mean ± SD)	Ear/Facial domain (mean ± SD)	Sleep domain (mean ± SD)	Function domain (mean ± SD)	Emotion domain (mean ± SD)
Jacobs et al., 2025	Baseline score: Tezepelumab 49.6 ± 20.1; Placebo 49.3 ± 19.6; LS Mean difference: week 28: −12.00; week 52: −11.08	Baseline score: Tezepelumab 2.7 ± 1.0; Placebo 2.6 ± 0.9; LS Mean difference: week 28: −0.5; week 52: −0.60	Baseline score: Tezepelumab 1.5 ± 1.1; Placebo 1.4 ± 1.0; LS Mean difference: week 28: −0.37; week 52: −0.31	Baseline score: Tezepelumab 2.4 ± 1.1; Placebo 2.5 ± 1.2; LS Mean difference: week 28: −0.67; week 52: −0.67	Baseline score: Tezepelumab 2.3 ± 1.2; Placebo 2.5 ± 1.2; LS Mean difference: week 28: −0.78; week 52: −0.53	Baseline score: Tezepelumab 1.8 ± 1.2; Placebo 1.8 ± 1.3; LS Mean difference: week 28: −0.51; week 52: −0.30
Lipworth et al., 2025	Baseline: 68.7 ± 18.4 LS mean difference week 52: −27.26;					
Laidlaw et al., 2023	Baseline score: Tezepelumab 49.6 ± 20.1; Placebo 49.3 ± 19.6; LS Mean change week 52: −10.58; Tezepelumab: −21.06 ± 2.50; Placebo: −10.48 ± 2.56	Baseline score: Tezepelumab 2.7 ± 1.0; Placebo 2.6 ± 0.9; LS Mean change week 52: −0.52; Tezepelumab: −1.08 ± 0.13; Placebo: −0.56 ± 0.13	Baseline score: Tezepelumab 1.5 ± 1.1; Placebo 1.4 ± 1.0; LS Mean change week 52: −0.21 Tezepelumab: −0.47 ± 0.14; Placebo: −0.26 ± 0.14	Baseline score: Tezepelumab 2.4 ± 1.1; Placebo 2.5 ± 1.2; LS Mean change week 52: −0.72; Tezepelumab: −1.27 ± 0.14; Placebo: −0.55 ± 0.14	Baseline score: Tezepelumab 2.3 ± 1.2; Placebo 2.5 ± 1.2; LS Mean change week 52: −0.61; Tezepelumab: −1.12 ± 0.14; Placebo: −0.51 ± 0.14	Baseline score: Tezepelumab 1.8 ± 1.2; Placebo 1.8 ± 1.3; LS Mean change week 52: −0.36; Tezepelumab: −0.69 ± 0.15; Placebo: −0.33 ± 0.15

Table 2B Other outcomes							
Author (year)	ACQ-6 score (mean ± SD)	AQLQ (S) + 12 score (mean ± SD)	ASD score (mean ± SD)	Nasal congestion score (mean ± SD)	Nasal Polyp Score (mean ± SD)	Lund-Mackay score (mean ± SD)	Pre-bronchodilator FEV1 (mean ± SD)
Jacobs et al., 2025	Baseline: No history of CRSwNP: 2.80 ± 0.81; History of CRSwNP: 2.85 ± 0.84; LS Mean difference, week 52: No history of CRSwNP: −0.27; History of CRSwNP: −0.63	Baseline: No history of CRSwNP: 38.4% >2; History of CRSwNP: 2.85 ± 0.84; LS Mean difference, week 52: No history of CRSwNP: −0.25; History of CRSwNP: −0.77	Baseline: No history of CRSwNP: 1.39 ± 0.69; History of CRSwNP: 1.42 ± 0.69; LS Mean difference, week 52: No history of CRSwNP: −0.32; History of CRSwNP: −0.08				Baseline: No history of CRSwNP: 1.83 ± 0.71; History of CRSwNP: 1.88 ± 0.71, LS Mean difference, week 52: No history of CRSwNP: 0.20; History of CRSwNP: 0.12
Lipworth et al., 2025	Baseline: 1.82 ± 1.16 LS mean difference week 52: −1.03			Baseline: 2.57 ± 0.51 LS mean difference week 52: −1.03	Baseline: 6.1 ± 1.2 LS mean difference week 52: −2.07	Baseline: 18.7 ± 3.8 LS mean difference week 52: −5.72	Baseline: 28.9 ± 0.89; LS mean difference week 52: −0.01
Laidlaw et al., 2023	Baseline: No history of CRSwNP: 2.80 ± 0.81; History of CRSwNP: 2.85 ± 0.84; LS Mean change week 52: −0.80; Tezepelumab: −2.87 ± 0.82; Placebo: −2.78 ± 0.84	Baseline: No history of CRSwNP: 38.4% >2; History of CRSwNP: 2.85 ± 0.84; LS Mean change week 52: −0.99; Tezepelumab: −3.84 ± 1.02; Placebo: −3.83 ± 0.90	Baseline: No history of CRSwNP: 1.39 ± 0.69; History of CRSwNP: 1.42 ± 0.69; LS Mean change week 52: −0.45; Tezepelumab: −1.41 ± 0.72; Placebo: −1.45 ± 0.72				Baseline: No history of CRSwNP: 1.83 ± 0.71; History of CRSwNP: 1.95 ± 0.75; LS Mean difference, week 52 No history of CRSwNP: 0.21; History of CRSwNP: 0.12

Jacobs JS et al.^17^ conducted a post hoc analysis within the phase 3 NAVIGATOR trial (NCT03347279) involving 1059 patients (aged 12–80 years) with severe, uncontrolled asthma, randomized to Tezepelumab 210 mg or placebo subcutaneously every 4-weeks for 52-weeks. They focused, in particular, on 165 (15.6%) patients who had a history of CRSwNP. Tezepelumab treatment resulted in sustained improvements versus placebo in SNOT-22 total score throughout the 52-week study period [least-squares mean difference (95% Confidence Interval) −11.08 (−17.80, −4.35)]. Tezepelumab improved all five SNOT-22 domain scores (sleep, nasal, function, ear/facial, and emotion) and the five SNOT-22 item scores of most clinical interest (decreased sense of smell/taste, nasal blockage, reduced productivity, waking up tired, and cough).

Laidlaw TM et al.^18^ assessed the efficacy of tezepelumab in 118 patients with severe asthma and comorbid Nasal Polyps (NPs) within the 2-years preceding randomization in the NAVIGATOR trial. At week 52, tezepelumab improved lung function, asthma control, and HRQoL versus placebo in patients with and without NPs. Tezepelumab reduced SNOT-22 total scores (least-squares mean difference versus placebo [95% CI]) in patients with NPs at 28-weeks (−12.57 points [−19.40, −5.73]) and 52-weeks (−10.58 points [−17.75, −3.41]). At week-52, tezepelumab reduced blood eosinophil counts and FeNO, IgE, IL-5, IL-13, EDN, and MMP-10 levels compared to placebo, regardless of NP status.

Lipworth BJ et al.^10^ investigated the efficacy of tezepelumab in 408 adult patients with severe CRSwNP in their phase 3 WAYPOINT trial (NCT04851964). In total, 203 patients were assigned to receive Tezepelumab, and 205 were assigned to receive a placebo. At week 52, the patients who received tezepelumab had significant improvements in the total nasal-polyp score (mean difference vs. placebo, −2.07; 95% Confidence Interval [95% CI], −2.39 to −1.74) and the mean nasal-congestion score (−1.03; 95% CI, −1.20 to −0.86) (p < 0.001 for both scores). Tezepelumab significantly improved the loss-of-smell score (mean difference vs. placebo, −1.00; 95% CI, −1.18 to −0.83), SNOT-22 total score (−27.26; 95% CI, −32.32 to −22.21), Lund-Mackay score (−5.72; 95% CI, −6.39 to −5.06), and total symptom score (−6.89; 95% CI, −8.02 to −5.76) (p < 0.001 for all scores). Surgery for nasal polyps was indicated in significantly fewer patients in the tezepelumab group (0.5%) than in the placebo group (22.1%) (hazard ratio, 0.02; 95% CI, 0.00 to 0.09); there was significantly less use of systemic glucocorticoids with Tezepelumab (5.2%) than with placebo (18.3%) (Hazard Ratio = 0.12; 95% CI, 0.04 to 0.27) (p < 0.001 for both time-to-event analyses).

## Discussion

In recent years, several innovative biological therapies have emerged as effective treatment options for patients with Chronic Rhinosinusitis with Nasal Polyps (CRSwNP. Among the most widely studied and utilized agents are omalizumab (anti-IgE), mepolizumab and benralizumab (anti-IL-5 and anti-IL-5R, respectively), and dupilumab (anti-IL-4Rα, blocking both IL-4 and IL-13 signaling). These biologics have demonstrated significant benefits in reducing the frequency of disease exacerbations, improving sinonasal symptoms, enhancing pulmonary function, and ultimately contributing to better overall disease control and quality of life. Their introduction has marked a substantial advance in the management of Type 2 (T2) inflammatory airway diseases.

However, despite these therapeutic improvements, a substantial proportion of patients continue to experience suboptimal CRSwNP control, persistent symptoms, and frequent exacerbations. One potential explanation for this residual disease burden is the narrow specificity of current biologics, which typically target single downstream mediators within the T2 inflammatory cascade. As a result, they may be insufficiently effective in patients with complex or overlapping inflammatory profiles, including mixed or non-eosinophilic endotypes. This limitation underscores the need for novel therapeutic approaches that can provide broader immunological coverage and more robust modulation of upstream inflammatory signals.

Tezepelumab is a first-in-class human monoclonal antibody that addresses this need by targeting TSLP, an epithelial cell-derived alarmin that plays a central role in the initiation and amplification of airway inflammation.^12^ Released in response to environmental insults such as allergens, microbes, pollutants, or mechanical injury, TSLP acts early in the inflammatory cascade to activate dendritic cells, which subsequently prime naïve T cells toward Th2 differentiation. This leads to downstream production of key cytokines (IL-4, IL-5, IL-13) and recruitment of effector cells such as eosinophils, basophils, and mast cells. By blocking TSLP signalling at its receptor complex, tezepelumab exerts upstream inhibition of the entire cascade, with potential benefits across both T2 and non-T2 inflammatory pathways.^3^

Administered via subcutaneous injection once every four weeks, tezepelumab has demonstrated encouraging results in several clinical trials for severe asthma, and more recently, in studies focused on CRSwNP. Despite these promising findings, to date, there has been no comprehensive effort to synthesize the available evidence on tezepelumab’s efficacy and safety in this specific patient population. The current study aims to fill this gap by presenting, to the best of our knowledge, the first systematic review and meta-analysis that aggregates data from all available Phase 2 and Phase 3 randomized controlled trials comparing tezepelumab with placebo.

This comprehensive evaluation aims to provide an in-depth analysis of tezepelumab’s clinical performance in terms of symptom control, reduction in polyp burden, biomarker modulation, and safety outcomes. By consolidating the current evidence, this work not only offers clinicians a clearer understanding of tezepelumab’s therapeutic potential but also identifies knowledge gaps and priorities for future research. Given the increasing complexity of inflammatory airway disease management and the emergence of precision medicine approaches, this review represents a timely and relevant contribution to the evolving landscape of biologic therapies in CRSwNP.

The reviewed studies highlight the effectiveness of tezepelumab in improving clinical outcomes for patients with severe asthma and comorbid CRSwNP. All authors reported improvements in SNOT-22 total scores, with Least Squares (LS) mean differences between treatment arms ranging from −11.08 to −27.26 at week 52. Benefits were also observed in all other SNOT domains, including sleep, nasal symptoms, and emotional well-being. Patients suffering from CRSwNP also exhibited greater improvements in asthma control (ACQ-6 = −0.63) compared to those without nasal polyps (0.27), suggesting that comorbid CRSwNP may identify a subgroup with particularly favourable responses to TSLP inhibition.^10^^,^^17^^,^^18^

Objective indicators of nasal disease severity consistently demonstrated substantial improvements across the reviewed studies. Notably, both Lipworth et al. and Laidlaw et al. reported significant reductions in Lund-Mackay scores (e.g., −5.72 in Lipworth et al.), which strongly suggest that tezepelumab has a meaningful effect on underlying sinonasal inflammation. Among the most clinically impactful findings was tezepelumab’s steroid-sparing capacity: Lipworth et al. documented a marked reduction in systemic corticosteroid use in the treatment group (5.2%) compared to placebo (18.3%). Furthermore, the nearly complete elimination of nasal polyp surgeries (0.5% vs. 22.1%) highlights the potential of tezepelumab not only to control symptoms but also to modify the disease course of CRSwNP, potentially delaying or even preventing the need for surgical intervention.^10^

Tezepelumab’s unique mechanism of action offers distinct advantages over existing biologic therapies for CRSwNP. While agents such as omalizumab (anti-IgE), mepolizumab (anti-IL-5), and dupilumab (anti-IL-4Rα/IL-13) selectively block downstream elements of the type 2 inflammatory pathway, tezepelumab targets Thymic Stromal Lymphopoietin (TSLP), an epithelial-derived alarmin released in response to diverse stimuli such as allergens, pathogens, or mechanical injury. TSLP initiates and amplifies inflammatory responses by activating dendritic cells, promoting the differentiation of naïve T-cells into Th2 cells, and ultimately driving the recruitment of eosinophils, basophils, mast cells, and other inflammatory mediators.^3^^,^^11^

By inhibiting TSLP upstream in the inflammatory cascade, tezepelumab offers a more comprehensive immunomodulatory effect, potentially addressing both type 2 and non-type 2 endotypes. This upstream action may explain its efficacy in patients with mixed or non-eosinophilic inflammation, populations that often demonstrate suboptimal responses to existing biologics.^11^ Moreover, improvements in both the Nasal Polyp Score (NPS) and radiologic findings (Lund-Mackay score) suggest structural changes beyond symptomatic relief, indicating a true disease-modifying effect.

The ability to reduce oral corticosteroid dependence and surgical intervention rates further strengthens the argument for tezepelumab as a robust therapeutic option. Its favorable safety profile, coupled with consistent efficacy across multiple clinical trials, reinforces its emerging role in the treatment of severe CRSwNP, especially in patients with inadequate responses to current standards of care.

Nonetheless, this study is not without limitations. The number of available Randomized Controlled Trials (RCTs) remains limited, and the analysis was constrained to data reported in peer-reviewed publications and registered at ClinicalTrials.gov. While pooled data on outcomes such as FEV1, blood eosinophil count, FeNO, total serum IgE, exacerbation rates, and asthma-related quality of life were assessable, subgroup analyses to explore sources of heterogeneity could not be performed due to insufficient data granularity. Furthermore, the follow-up durations in all included studies were capped at 52-weeks, leaving long-term efficacy and safety largely unexplored.

Future investigations should address these gaps through extended follow-up studies and the collection of real-world data. Additional trials should investigate the role of tezepelumab in various inflammatory airway diseases, including Chronic Obstructive Pulmonary Disease (COPD), allergic rhinitis, and Aspirin-Exacerbated Respiratory Disease (AERD), to fully elucidate its therapeutic potential. The potential for early intervention to modify disease progression, reduce healthcare utilization, and improve long-term outcomes remains a critical area for further research.

### Limitations and future perspectives

Despite these encouraging results, significant gaps remain in the current literature.

The most prominent limitation is the constrained evidence base. Our review identified only three eligible studies, two of which were derived from the same pivotal clinical trial program (NAVIGATOR), and, notably, just one randomized controlled trial has specifically addressed patients with CRSwNP. The lack of high-quality, targeted evidence greatly limits the generalizability of the findings. Moreover, there is a paucity of real-life studies, which currently makes it difficult to assess the true effectiveness and applicability of Tezepelumab in routine practice settings. Furthermore, the follow-up duration in these trials was capped at 52-weeks, leaving long-term safety, efficacy, and durability of response largely unexplored beyond the first year. Another critical issue is the need for improved patient selection: identifying which subgroups of CRSwNP patients are most likely to benefit from TSLP inhibition is essential to optimize therapeutic outcomes.

Ultimately, there is a pressing need to identify predictive biomarkers that enable clinicians to anticipate treatment responses. This would support a more personalized and targeted therapeutic approach, ultimately improving patient care and cost-effectiveness. Therefore, heterogeneity in reporting methodologies and assessment tools used in the included studies prevented a formal meta-analysis. Future research should aim to:

- Conduct long-term extension studies and collect real-world data to validate these initial findings to better understand the role of tezepelumab in CRSwNP management.
- Define clear criteria for patient stratification, identifying clinical or biological markers able to predict a superior response to tezepelumab compared to other biologics.
- Identify predictive biomarkers of treatment success to enhance personalized and cost-effective treatment strategies.

## Conclusion

Tezepelumab represents a promising and well-tolerated therapeutic option for comorbid and non-comorbid CRSwNP. By targeting TSLP, an upstream epithelial cytokine, tezepelumab suggests the potential for a broader immunomodulatory effect. Unlike other biologics that target single downstream mediators, such as IL-5, IL-4/13, or IgE, inhibiting the key alarmin may modulate the very beginning of the inflammatory cascade, potentially covering a wider range of inflammatory pathways. This unique mechanism of action enables potential benefits across a wider range of asthma endotypes, including those less responsive to traditional biologics. While current clinical trials have demonstrated encouraging results regarding its efficacy and safety profile, further large-scale, real-world studies and long-term RCTs are essential to validate these findings, assess durability of response, and better define its role within personalized treatment algorithms. Ongoing research should also explore its impact on quality of life, healthcare utilization, and disease progression to fully establish tezepelumab’s place in the management of severe asthma and related comorbidities.

## ORCID ID

Eugenio de Corso: 0000-0001-5761-7018

Domiziana Nardelli: 0009-0007-0572-8972

Jacopo Galli: 0000-0003-1599-4573

Manuele Casale: 0009-0003-7255-4619

## Funding

The authors declare no specific funding.

## Data availability statement

The authors declare that all data are available in repository.

## Declaration of competing interest

The authors declare no conflicts of interest.
